# Effect of *DNMT3A* polymorphisms on CpG island hypermethylation in gastric mucosa

**DOI:** 10.1186/s12881-020-01142-7

**Published:** 2020-10-16

**Authors:** Hikaru Takano, Tomoyuki Shibata, Masakatsu Nakamura, Naoko Sakurai, Tasuku Hayashi, Masafumi Ota, Tomoe Nomura-Horita, Ranji Hayashi, Takeo Shimasaki, Toshimi Otsuka, Tomomitsu Tahara, Tomiyasu Arisawa

**Affiliations:** 1 Department of Gastroenterology, Kanazawa Medical University, 1-1 Daigaku, Uchinada-machi, Ishikawa 920-0293 Japan; 2 Department of Gastroenterology, Fujita Health University, 1-98 Dengakugakubo, Kutsukake-cho, Toyoake, 470-1192 Japan; 3 Department of Gastroenterology and Hepatology, Kansai Medical University, 2-5-1 Shin-machi, Hirakata, Osaka, 573-1010 Japan

**Keywords:** *DNMT3A*, Genetic polymorphism, CpG island, Hypermethylation, Gastric mucosa

## Abstract

**Background:**

CpG methylation of tumor suppressor genes occurs in the early stage of carcinogenesis. Detecting risk factors for aberrant CpG methylation is clinically important for predicting cancer development. DNA methyltransferase (DNMT) 3a is considered to play critical roles in the DNA methylation process during pathogenesis. In this study, we evaluated the association between *DNMT3A* polymorphisms (rs6733868 and rs13428812) and CpG methylation status in non-cancerous gastric mucosa.

**Methods:**

We determined the *DNMT3A* genotype and CpG methylation status of 4 genes (*p14*^*ARF*^, *p16*^*INK4a*^, *DAPK*, and *CDH1*) in 510 subjects without gastric cancer. *Helicobacter pylori* (HP) infection status was determined by the rapid urease test, urea breath test, speculum examination, or serum antibody test. We determined the *DNMT3A* genotype using polymerase chain reaction single-strand conformation polymorphism (PCR-SSCP). CpG methylation status was determined by methylation-specific polymerase chain reaction (MSP). When the methylated band was stronger than 10 ng/μL according to the DNA marker, we judged CpG island hypermethylation (CIHM) to be present. Associations between genotypes and susceptibilities were assessed by logistic regression analysis.

**Results:**

The minor allele frequencies of both polymorphisms (rs6733868 and rs13428812) were lower in the CpG methylated groups of each of the 4 genes (*p14*^*ARF*^, *p16*^*INK4a*^, *DAPK*, and *CDH1*). Using a dominant genetic model, rs6733868 was significantly associated with the hypermethylation of each gene, whereas rs13428812 was associated with the methylation of 3 genes (all except *p14*^*ARF*^). When low-CIHM was defined as 1 or 2 CpG islands methylated and high-CIHM was defined as 3 or more CpG islands methylated, carrying the minor allele of rs6733868 was associated with both decreased low- and high-CIHM, and that of rs13428812 also was associated with a decrease. Comparing low-CIHM with high-CIHM, carrying the minor alleles of rs6733868 or rs13428812 was related to decreased susceptibility to high-CIHM. In HP-infected subjects, carrying the minor alleles of rs6733868 or rs13428812 had a significantly greater association with decreased susceptibility to high-CIHM.

**Conclusions:**

Our study indicates that polymorphisms of *DNMT3A* are associated with the accumulation of gene methylation in gastric mucosa. Carrying the minor alleles of rs6733868 or rs13428812 inhibits aberrant gene methylations, which are typically enhanced by HP infection.

## Background

Gastric cancer is one of the most common and deadly malignancies in the world. In 2018, gastric cancer was ranked as the fifth most commonly diagnosed cancer globally, and gastric cancer deaths accounted for 8.2% of all cancer deaths. Gastric cancer is thus the third leading cause of death from cancer. Additionally, more than 50% of gastric cancers occur in eastern Asia [1]. Despite the declining trend in Japan due to insurance coverage to eradicate *Helicobacter pylori* (HP), gastric cancer remains a clinically significant malignancy, affecting 50,000 people annually. It has long been known that HP infection is a risk factor for gastric cancer [2, 3], and it has been suggested that three steps are involved in gastric cancer progression: HP infection, the development of a precancerous state of the stomach, and carcinogenesis [4]. Correa proposed an oncogenic sequence in which differentiated gastric cancer (intestinal type) develops from HP infection through chronic gastritis (atrophic gastritis, intestinal epithelialization) [5]. Therefore, it has been recognized that advanced atrophic gastritis and intestinal epithelialization are essential conditions of the precancerous stage of gastric cancer from both morphological and histological perspectives [6].

From a molecular biological perspective, however, Maekita et al. found that the accumulation of abnormal DNA methylation in the gastric mucosa is critical for the precancerous state [7]. We also obtained the same results [8]. Methylation at CpG islands is a key mechanism of gene silencing, and there are known aberrant methylations occurring in specific genes in a variety of cancers, including gastric cancer [9, 10]. Among the three known DNA methyltransferases (DNMTs), DNMT3a and DNMT3b are de novo DNMTs and are critical enzymes that cause dynamic DNA methylation during embryogenesis and pathogenesis [11]. In addition, overexpression of *DNMTs* has been observed in gastric carcinoma and non-neoplastic tissues susceptible to gastric carcinoma [12]. These facts suggest that CpG island hypermethylations (CIHM) caused by DNMTs might be crucial in the development of gastric cancer.

While HP infection is involved in the development of gastric cancer, the genetic constitution of an individual might also be involved, as gastric cancer does not occur in all HP-infected individuals. El-Omar and coworkers were the first to report that a genetic polymorphism of interleukin-1β is implicated in gastric cancer [13]. In our previous study, we demonstrated a relationship between gene polymorphisms of specific genes and gastric cancer susceptibility [14, 15]. Despite the involvement of rs1550117, a known representative polymorphism of *DNMT3A* reported in various carcinomas, its role in gastric cancer is still controversial, particularly in Japanese patients [16]. Recently, we found that rs6733868 C > G and rs13428812 A > G of *DNMT3A* are involved in HP infection, the progression of gastric mucosal atrophy, and gastric cancer susceptibility in a Japanese population [17]. However, the involvement of these *DNMT3A* gene polymorphisms in the accumulation of aberrant CpG methylation in the gastric mucosa has not been clarified.

We sought to elucidate the effects of *DNMT3A* polymorphisms rs6733868 and rs13428812 in the accumulation of CpG methylation in the gastric mucosa and how HP infection might impact the process.

## Methods

### Population samples

The study population comprised 510 subjects without cancer who attended the Endoscopy Center of Fujita Health University Hospital from January 2006 to December 2012. In all of the study participants, an upper gastrointestinal endoscopy was performed as a part of a health check-up, as a secondary examination following barium X-ray gastrography, or for any symptoms of abdominal discomfort. Of these, 402 were subjects recruited from our previous research [18]. Our exclusion criteria included: subjects with severe systemic diseases or malignancies of the stomach or other organs and any participant with a history of abdominal surgery or HP eradication. The study protocol was approved by the Ethics Committee of Fujita Health University School of Medicine, Japan, and written informed consent was obtained from all participants.

For each subject, biopsy samples were taken from the antrum at the time of endoscopy, and one part of each was immediately frozen and stored at − 80 °C until use. Peripheral blood was collected at the time of endoscopy, and serum was prepared and frozen at − 80 °C. HP infection was determined when at least one of the following tests was positive: 1) rapid urease test, 2) urea breath test, 3) speculum examination, or 4) serum antibody test. Two tumor suppressor genes with methylation associated with aging and HP infection (*p14*^*ARF*^ and *p16*^*INK4a*^, respectively), death-associated protein kinase (*DAPK*), and E-cadherin (*CDH1*) were selected as candidates for the evaluation of CIHM [7, 17, 18]. Both p14^ARF^ and p16^INK4a^ are translated from *CDKN2A* by alternative splicing [19, 20]. These four genes were selected because increased CpG island hypermethylation in these genes in non-neoplastic gastric mucosa has been shown to correlate with a higher risk of gastric cancer [21].

### Genotyping

Genomic DNA was extracted from a portion of the frozen samples using proteinase K. In 408 cases, genomic DNA was extracted from blood samples. Genotyping was performed by the previously described polymerase chain reaction (PCR)-single-strand conformation polymorphism (SSCP) method [22]. The following primer sets were used: for rs6733868: forward, 5′-ctagctagcgggagtcgctgtc-3′ and reverse, 5′-ctcctggctgtgaagcggaag-3′; for rs13428812: forward, 5′-ccccatcatgtcagataccctctg-3′ and reverse, 5′-ccttcctaggggacacccttctatt-3′.

PCR was performed using EX Taq HS (Takara Bio, Shiga, Japan), adding 0.1 μg of genomic DNA to 20 μL of buffer, denaturing at 95 °C for 3 min, followed by 35 cycles of 15 s at 96 °C, 30 s at 61 °C, and 30 s at 72 °C, and a 5-min final extension at 72 °C. The same PCR conditions were used for rs6733868 and rs13428812. Then, 2 μL of the PCR product was treated in 10 μL of formamide for 5 min at 90 °C and denatured to single strands. SSCP was performed with the Gene Phor DNA separation system using the Gene Gel Excel 12.5/24 kit (GE Health Care Bio-Sciences AB, Stockholm, Sweden) at a constant temperature of 18 °C, and the denatured bands were detected using a DNA silver staining kit (GE Health Care Bio-Sciences AB).

### Bisulfite reaction and methylation-specific PCR (MSP) methods

To detect DNA methylation, genomic DNA extracted from biopsy tissues was treated with sodium bisulfite using a BislFast DNA Modification Kit for Methylated DNA Detection (Toyobo, Osaka, Japan). The methylation-specific PCR (MSP) reaction was performed as previously described [8, 18]. The primer sets used are shown in Table 1. Using EX Taq HS (Takara Bio, Shiga, Japan), PCR was performed for 0.1 μg of bisulfite-modified DNA in 20 μL of buffer with an initial denaturing step of 5 min at 95 °C, followed by 33–35 cycles of 30 s denaturing at 95 °C, 1 min annealing at 57–69 °C, and 1 min extension at 72 °C, and a final 5-min extension step at 72 °C. 2.5% agarose gel electrophoresis was performed using 10 μL of PCR product, stained with ethidium bromide, and visualized by UV illumination. The presence of CIHM was judged when the signal of the electrophoresis-separated positively-methylated band was stronger than that of the size marker (10 ng/μL: 100 bp DNA Ladder; Takara Bio), regardless of the presence of unmethylated bands [23].

**Table 1:** Primer sets used for methylation-specific polymerase chain reaction

gene name	methylated forward	methylated reverse	product size
*p14*^*ARF*^	5′-gtgttaaagggcggcgtagc-3’	5′-aaaaccctcactcgcgacga-3’	122 bp
*p16*^*INK4a*^	5′-ttattagagggtggggcggatcgc-3’	5′-gaccccgaaccgcgaccgtaa-3’	150 bp
*DAPK*	5′-ggatagtcggatcgagttaacgtc-3’	5′-ccctcccaaacgccga-3’	98 bp
*CDH1*	5′- ttaggttagagggttatcgcgt-3’	5′-taactaaaaattcacctaccgac-3’	115 bp
	unmethylated forward	unmethylated reverse	
*p14*^*ARF*^	5′-tttttggtgttaaagggtggtgtagt-3’	5′-cacaaaaaccctcactcacaacaa-3’	132 bp
*p16*^*INK4a*^	5′-ttattagagggtggggtggattgt-3’	5′-caaccccaaaccacaaccataa-3’	151 bp
*DAPK*	5′-ggaggatagttggattgagttaatgtt-3’	5′-caaatccctcccaaacaccaa-3’	106 bp
*CDH1*	5′- taattttaggttagagggttattgt-3’	5′-cacaaccaatcaacaacaca-3’	97 bp

### Statistical analysis

Hardy-Weinberg equilibria were assessed by the χ^2^ test. Mean age was expressed as mean ± SD and analyzed using Student’s *t* test. HP infection rate and sex ratio were compared using Fisher’s exact test. Genotype distribution and allele frequency were also assessed using Fisher’s exact test. Odds ratios (OR) and 95% confidence intervals (CI) for the extent of genotype involvement in DNA methylation were calculated using logistic regression analysis adjusted for sex, age, and HP infection status. The relationship between the number of genes with CpG methylation and genotype or HP positivity was assessed by ANCOVA. All analyses were considered significant with *p* < 0.05. Stata software (version 13; StataCorp LP, College Station, TX, USA) was used for statistical processing.

## Results

### Characteristics of the subjects and allelic frequencies in each methylated population

The background and distribution of genotypes of the subjects, including CpG island methylation status of each of the four genes, are shown in Table 2. In the 510 subjects, the distribution for the *DNMT3A* variants was as follows: rs6733868: CC = 212, CG = 224, and GG = 74; rs13428812: AA = 313, AG = 166, and GG = 31, both meeting Hardy-Weinberg equilibrium (*p* = 0.25 and *p* = 0.16, respectively), and these distributions did not differ from the data reported by HapMap-JPT (*p* = 0.51 and *p* = 0.20, respectively). The CpG methylated subjects for the genes (*p14*^*ARF*^, *p16*^*INK4a*^, *DAPK*, and *CDH1*) were 167, 134, 252, and 192, respectively. The mean age in the *p14*^*ARF*^-methylated group was significantly higher than that in the unmethylated group, whereas no significant difference was found in the other three genes. The male/female ratio was not significantly different among methylated and unmethylated groups in all four genes. The ratio of HP positivity was significantly higher in the methylated group than in the unmethylated group in all four genes. In both gene polymorphisms, the minor allele frequency in the methylated groups tended to be lower.

**Table 2:** Characteristics of the subjects and allelic frequency in CpG methylated population of each genes

	overall	*p14*^*ARF*^-M	*p16*^*INK4a*^-M	*DAPK*-M	*CDH1*-M
number of sample	510	167	134	252	192
mean age ± SD	60.5 ± 13.7	62.9 ± 13.7^a^	61.2 ± 11.8	61.5 ± 13.6	60.3 ± 13.0
male: female	297: 213	91: 76	78: 56	150: 102	117: 75
HP infection status	321/510	117/167^b^	111/134^c^	186/252^c^	148/192^c^
(rs6733868 C > G)					
CC	212	81^d^	69^e^	122^f^	100^g^
CG	224	61	49	112^c^	70
GG	74	25	16	18	22
G allele freqency	36.5%	33.2%	30.2%^h^	29.4%^c^	29.7%^i^
(rs13428812 A > G)					
AA	313	111	97^j^	169^k^	145^c^
AG	166	47	28	73	37
GG	31	9	9	10	10
G allele freqency	22.4%	19.5%	17.2%^l^	18.5%^m^	14.8%^c^

-*M* Methylated

a: *p* = 0.0046, b: *p* = 0.019, c: *p* < 0.0001, d: *p* = 0.028, e: *p* = 0.0079, f: *p* = 0.0022, g: *p* = 0.0002, h: *p* = 0.015, i: *p* = 0.0005,

j: *p* = 0.0027, k: *p* = 0.011, l: *p* = 0.017, m: *p* = 0.0034 vs. unmathylated group of each gene

### Association between *DNMT3A* polymorphisms and CpG hypermethylation of each gene

For the polymorphism rs6733868, the methylation of all four genes under study (*p14*^*ARF*^, *p16*^*INK4a*^, *DAPK*, and *CDH1*) showed significant involvement as revealed by regression analysis using a dominant genetic model adjusted for sex, age, and HP infection status (Table 3). In addition, a recessive genetic model also showed the significant involvement of *DAPK* methylation. For the polymorphism rs13428812, the dominant genetic model showed a significant association with methylation in three genes, all except *p14*^*ARF*^. In contrast, the recessive genetic model showed no significant involvement of methylation in any of the four genes (Table 4).

**Table 3:** Association between *DNMT3A* rs6733868 and CpG methylation of each genes

genotype	*p14*^*ARF*^-UM (343)	*p14*^*ARF*^-M (167)	adjusted OR* (95%CI); *p* value	
CC	131	81	reference	reference
CG	163	61		0.67 (0.46–0.98); 0.038
GG	49	25	1.07 (0.629–1.82); 0.80	
genotype	*p16*^*INK4a*^-UM (376)	*p16*^*INK4a*^-M (134)	adjusted OR* (95%CI); *p* value	
CC	143	69	reference	reference
CG	175	49		0.60 (0.40–0.91); 0.015
GG	58	16	0.78 (0.42–1.44); 0.42	
genotype	*DAPK*-UM (258)	*DAPK*-M (252)	adjusted OR* (95%CI); *p* value	
CC	90	122	reference	reference
CG	112	112		0.59 (0.41–0.85); 0.0050
GG	56	18	0.28 (0.15–0.49); < 0.0001	
genotype	*CDH1*-UM (318)	*CDH1*-M (192)	adjusted OR* (95%CI); *p* value	
CC	112	100	refernce	reference
CG	154	70		0.52 (0.36–0.75); 0.0005
GG	52	22	0.70 (0.40–1.20); 0.19	

-UM: unmethylated; −M: methylated

*: adjusted for gender, age and HP infection status

**Table 4:** Association between *DNMT3A* rs13428812 and CpG methylation of each genes

genotype	*p14*^*ARF*^-UM (343)	*p14*^*ARF*^-M (167)	adjusted OR* (95%CI); *p* value	
AA	202	111	reference	reference
AG	119	47		0.77 (0.52–1.14); 0.19
GG	22	9	0.93 (0.41–2.10); 0.87	
genotype	*p16*^*INK4a*^-UM (376)	*p16*^*INK4a*^-M (134)	adjusted OR* (95%CI); *p* value	
AA	216	97	reference	reference
AG	138	28		0.55 (0.35–0.85); 0.0078
GG	22	9	1.44 (0.62–3.37); 0.40	
genotype	*DAPK*-UM (258)	*DAPK*-M (252)	adjusted OR* (95%CI); *p* value	
AA	144	169	reference	reference
AG	93	73		0.66 (0.46–0.96); 0.030
GG	21	10	0.53 (0.240–1.17); 0.12	
genotype	*CDH1*-UM (318)	*CDH1*-M (192)	adjusted OR* (95%CI); *p* value	
AA	168	145	reference	reference
AG	129	37		0.37 (0.25–0.56); < 0.0001
GG	21	10	0.90 (0.40–2.00); 0.79	

-*UM* Unmethylated; −*M* Methylated

*: adjusted for gender, age and HP infection status

### Characteristics and allelic frequencies of the subjects by the number of methylations per gene

**Fig. 1:**
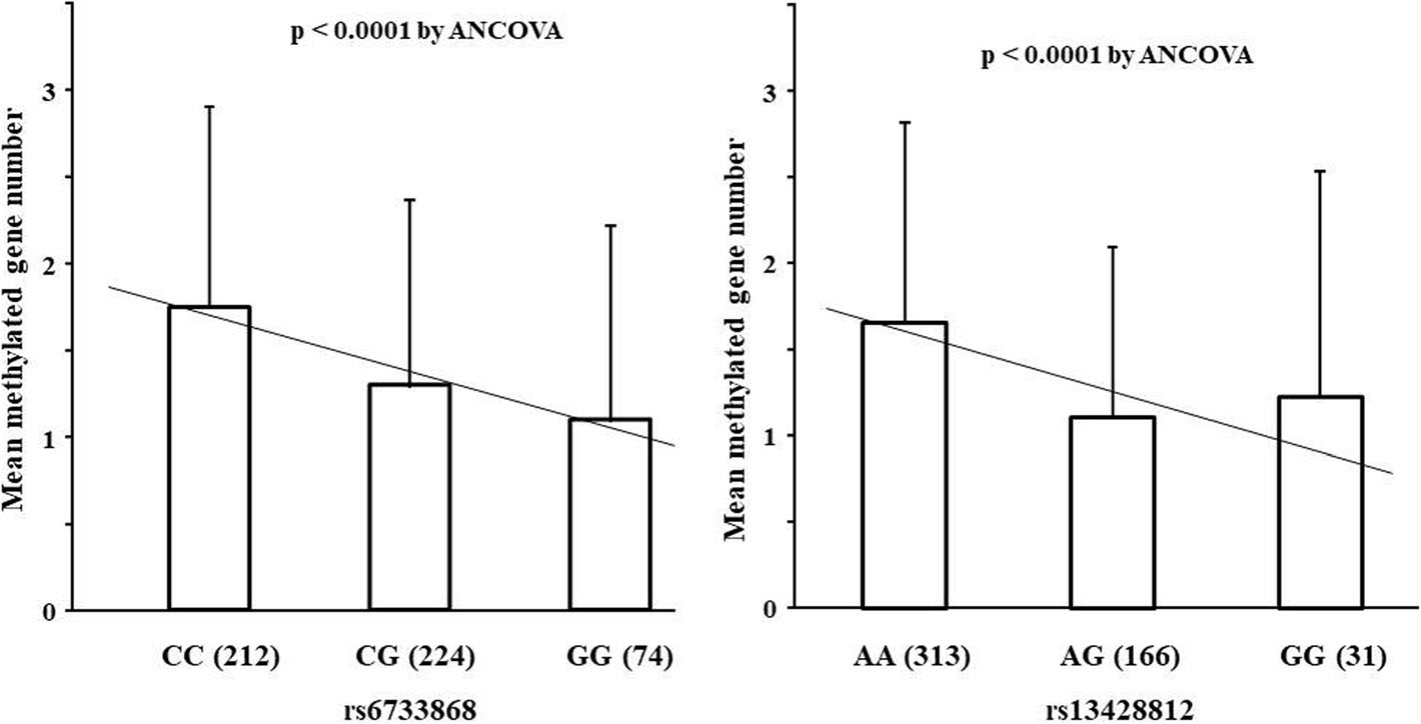
Correlation of genotypes with the number of genes with CpG methylation. There was a significant inverse correlation between minor allele number of both polymorphisms (rs6733868 and 13,428,812) and the number of genes with CpG methylation (*p* < 0.0001 by ANCOVA)

The distribution of background and genotype according to the number of methylations among the four genes were compared with a reference group (without methylated genes) (Table 5). The mean age of subjects tended to be significantly higher in those with methylated genes among the four genes selected for this study. Also, the rate of HP infection increased with the number of methylated genes and was considerably higher for all degrees of methylation. In addition, HP positivity was significantly correlated with the increased number of methylated genes (*p* < 0.0001 by ANCOVA). For the polymorphism rs6733868, the frequency of the CC genotype was significantly higher in the methylated group; conversely, the frequency of the GG genotype was lower, and the frequency of the minor allele tended to decrease with the number of methylated genes. Similarly, for the polymorphism rs13428812, the AA genotype tended to be significantly more frequent in the methylated group, while the GG genotype was less frequent, and the minor allele frequency tended to decrease with the number of methylated genes. An inverse correlation between minor allele number for both polymorphisms (rs6733868 and 13,428,812) and the number of CpG methylated genes was found (Fig. 1).

**Table 5:** Characteristics and allelic frequency of the subjects by number of methylated genes

number of methylated gene	0	1	2	3	4
number of sample	123	156	131	75	25
mean age ± SD	59.8 ± 14.9	59.9 ± 13.3	59.5 ± 14.1	61.9 ± 12.3	68.2 ± 10.3^a^
male: female	66: 57	100: 56	71: 60	46: 29	14: 11
HP infection status	53/123	89/156^b^	89/131^c^	67/75^c^	23/25^c^
(rs6733868 C > G)					
CC	36	56	61^d^	44^c^	15^e^
CG	57	82	53	24	8
GG	30	18^f^	17^g^	7^h^	2
G allele freuqency	47.6%	37.8%^i^	33.2%^j^	25.3%^c^	24.0%^k^
(rs13428812 A > G)					
AA	57	93^l^	82^m^	61^c^	20^n^
AG	53	57	43	10	3
GG	13	6^o^	6	4	2
G allele frequency	32.1%	22.1%^p^	21.0%^q^	12.0%^c^	14.0%^r^

a: *p* = 0.0082, b: *p* = 0.016, c: *p* < 0.0001, d: *p* = 0.0065, e: *p* = 0.0051, f: *p* = 0.0063, g: *p* = 0.023, h: *p* = 0.0085,

i: *p* = 0.025, j: *p* = 0.0011, k: *p* = 0.0027, l: *p* = 0.030, m: *p* = 0.012, n: *p* = 0.0021, o: *p* = 0.032, p: *p* = 0.0091,

q: *p* = 0.0048, r: *p* = 0.010 vs. 0 group

### Association between *DNMT3A* polymorphisms and the number of methylated genes

We performed regression analysis using a dominant genetic model adjusted for sex, age, and HP infection status in three groups: a non-CIHM group without methylated genes, a low-CIHM group with one or two methylated genes, and a high-CIHM high with three or more methylated genes (Table 6). For the polymorphism rs6733868, carrying the minor allele was significantly associated with a low risk of methylation in both the low-CIHM and high-CIHM groups compared with the non-CIHM group. By comparing the low- and high-CIHM groups, a significant association was observed for carriers of the minor allele with a reduced risk of methylation. Similar results were also obtained with the rs13428812 polymorphism.

**Table 6:** Association between *DNMT3A* polymorphisms and number of methylated genes in the whole population and in patients infected with HP

	Whole population				Patients infected with HP			
	Allelic variants			OR (95% C.I.); *p* value	Allelic variants			OR (95% C.I.); *p* value
rs6733868	CC	CG	GG		CC	CG	GG	
number of methylated genes								
0 (123)	36	57	30	reference	17	22	14	reference
1 or 2 (287)	117	135	35	0.59 (0.37–0.94); *p* = 0.026	69	89	20	0.74 (0.38–1.42); *p* = 0.36
3 or 4 (100)	59	32	9	0.34 (0.18–0.64); *p* < 0.0001	56	26	8	0.26 (0.12–0.55); *p* = 0.0004
	Allelic variants			OR (95% C.I.); *p* value	Allelic variants			OR (95% C.I.); *p* value
rs13428812	AA	AG	GG		AA	AG	GG	
number of methylated genes								
0 (123)	57	53	13	reference	26	21	6	reference
1 or 2 (287)	175	100	12	0.55 (0.36–0.85); *p* = 0.0073	107	67	4	0.61 (0.32–1.13); *p* = 0.12
3 or 4 (100)	81	13	6	0.22 (0.11–0.44); *p* < 0.0001	74	11	5	0.18 (0.083–0.41); *p* < 0.0001

by logistic regression analysis after adjustment for age, gender and HP infection status

(): number of subjects

In 321 (*n* = 510; 62.9%) HP-infected subjects, no significant association was noted with gene polymorphisms regarding methylation in the low-CIHM and non-CIHM groups (Table 6). In contrast, there was a strong and significant association in the high-CIHM group, indicating that carrying the minor allele for both gene polymorphisms (rs6733868 and rs13428812) was associated with a significant suppression of high-frequency CpG methylation.

## Discussion

DNMTs play an important role in DNA methylation and establish methylation patterns on CpG islands. Among the *DNMTs*, DNMT3a has a greater effect on de novo methylation than DNMT3b [8]. We previously demonstrated that *DNMT3A* polymorphisms (rs6733868 and rs13428812) are associated with the severity of gastric mucosal atrophy, which is accompanied by chronic inflammation and the subsequent development of gastric cancer [22]. However, whether these polymorphisms affect CpG island methylation as a precancerous condition has not been revealed. In this study, we examined whether either gene polymorphism was associated with the accumulation of methylation of CpG islands in the gastric mucosa. The results showed that the minor allele frequencies of rs6733868 (C > G) and rs13428812 (A > G) were significantly reduced as the number of methylations of the CpG islands of the four genes examined increased. Consistent with the fact that CpG methylation accumulates during inflammation and aging, we observed an increase in older subjects and with HP infection rates as the number of methylated CpGs increased in our study population. However, regression analysis after adjustment for confounding factors showed a strong and significant association of both gene variants, suggesting that *DNMT3A* gene polymorphism is an independent factor in the accumulation of methylation of gastric mucosal CpG islands of the studied genes. Although the *DNMT3A* polymorphism has been reported to be associated with gastric cancer, HP infection, and gastric mucosal atrophy [16, 22, 23], its association with methylation of gastric mucosa genes has not been clear. Our current study has revealed this association for the first time.

To assess the degree of CpG island methylation, we selected the CpG sites of four genes (*p14*^*ARF*^, *p16*^*INK4a*^, *DAPK*, and *CDH1*), because we previously reported that increased CpG island hypermethylation in these four genes of the non-neoplastic gastric mucosa correlates with a higher risk of gastric cancer [21]. The p14^ARF^ and p16^INK4a^ proteins are encoded by *CDKN2A*; these proteins act on the p53 and pRb pathways, respectively, to negatively regulate the cell cycle [19, 20]. The loss of function due to methylation or deletion of *CDKN2A* has been observed in many cancers [24]. Additionally, the importance of *DAPK* and *CDH1* in cancer has been revealed [25, 26]. Because these four genes play an important role in carcinogenesis, we could not exclude the possibility that methylation of these genes renders them silent, and hence, directly contributes to carcinogenesis. Nonetheless, since many reports describe CpG methylation of these genes in non-neoplastic areas [18, 21, 27], it is unlikely that their methylation contributes directly to carcinogenesis.

Global DNA methylation patterns in human cancer are altered by hypermethylation of the CpG islands and hypomethylation of the non-CpG parts [28]. The de novo DNMTs have also been implicated in this dynamic methylation early in tumor development [29]. We presumed that the methylation of these gene groups reflects the degree of change in global DNA methylation patterns, as they are confirmed to undergo methylation from the precancerous lesion stage. In the stomach, CIHM is associated with HP infection [7, 8, 17], the degree of gastritis [18], and the risk of carcinogenesis [23, 27]. Additionally, de novo DNMT expression is more highly enhanced in tumor and corneal tumor areas than in non-tumor areas [12]. These previous reports suggest that HP infection could induce de novo synthesis of DNMT genes in the stomach, with the subsequent methylation of CpG islands in genes, which in turn leads to carcinogenesis. In our current findings, the rate of HP infection increased with the increasing number of CpG methylated genes, and a significant relationship between both rs6733868 and rs13428812 gene polymorphisms and the number of CpG methylations was found only in the HP-infected subjects, but not in HP-uninfected subjects (data not shown). Notably, genetic polymorphisms are not the only regulators of protein expression. These gene polymorphisms might be of significance when there is an inducement by HP infection and the induction of gene expression triggered in DNMTs. However, HP infection does not directly induce DNMT mRNA [27]. Hmadcha et al. reported that the increase in DNMT activity by IL-1β is mediated by reactive oxygen species and nitric oxide [30]. Thus, it is likely that HP infection might have induced de novo DNMTs through this system, leading to methylation of CpG. Considering our results that minor alleles carrying the rs6733868 and rs13428812 gene polymorphisms correlated negatively with the accumulation of CpG methylation, we infer that these two types of gene polymorphisms are decreasing functional types.

There are several clinical limitations in this study. First, it was a retrospective study using samples collected at a single institution in Japan. The genetic polymorphisms examined in this study population satisfy the Hardy-Weinberg equilibrium, and the distribution of the genotypes is similar to that reported in the HapMap-JPT, which indicates that the population distribution is typical of Japanese citizens. However, a follow-up examination at another institution would be necessary. Second, there is no assurance that the validity of the four selected CpG gene sites studied represents changes in global DNA methylation patterns. Thus, based on the methylation status of CpG, the possibility of more methylation occurring at an earlier stage should be investigated. Finally, it is unclear how the genetic polymorphisms examined in this study might indeed affect the expression and function of *DNMT3a* protein. Fan et al. reported that rs1550117, an A > G variant in the *DNMT3A* gene promoter, affects protein expression and elevates *DNMT3a* expression, leading to the development of gastric cancer [31]. Therefore, we deduced that the minor alleles of the gene polymorphisms examined in this study might be of a hypofunctional type, but confirmation is required at the experimental level.

## Conclusions

Our study indicates that the polymorphisms of *DNMT3A* are associated with the accumulation of gene methylation in gastric mucosa. Carrying the minor allele of rs6733868 or rs13428812 inhibits aberrant gene methylations, especially under conditions of HP infection.

## Data Availability

All data generated during this study are included in this published article. The raw data analyzed during the current study are not publicly available due to risk of compromising individual privacy. The application and the written consent forms state that the data will only be available to the researchers within the project. For inquires on the data, researchers should first contact the owner of the database, Fujita Health University. Please contact the corresponding author with requests and for assistance with data requests.

## Abbreviations

HP: *Helicobacter pylori*
DNMT: DNA methyltransferase
CIHM: CpG island hyper methylations
*DAPK*: Death-associated protein kinase
*CDH1*: E-cadherin
PCR-SSCP: Polymerase chain reaction-single-strand conformation polymorphism
MSP: Methylation-specific PCR
UV: Ultraviolet
SD: Standard deviation
OR: Odds ratio
CI: Confidence interval
*CDKN2A*: Cyclin dependent kinase inhibitor 2A
